# The invisible body work of ‘last responders’ – ethical and social issues faced by the pathologists in the Global South

**DOI:** 10.1080/17441692.2022.2076896

**Published:** 2022-05-19

**Authors:** Halina Suwalowska

**Affiliations:** Nuffield Department of Population Health, Ethox Centre, Wellcome Centre for Ethics and Humanities, University of Oxford, Oxford, UK

**Keywords:** Pathologists, last responders, autopsy, ethical issues, body work

## Abstract

This paper utilises empirical data to explore the value of ‘body work’ performed by last responders charged with the duty of dead body management, with a focus on the Global South. While frontline staff work to save lives, little is known about the experiences and roles of those who care for the dead in global health in times of crises and even during normal times. This paper discusses ethical and socio-cultural challenges pathologists face in ‘working on the bodies of others’ while conducting any form of post-mortem procedures – necessary for ascertaining and recording the causes of death. Identifying and reporting the cause of death have significant public health benefits and provide closure for bereaved families. Despite the foregoing, the pathology field does not attract funding from governments or donors, and it is overlooked compared to other disciplines. Autopsy procedure bears social stigma – as it is associated with body mutilation and therefore disrespecting the dead; certain cultural beliefs or taboos about impurity and death persist, further raising some social and ethical tensions. As a result, the dearth of autopsy procedures contributes to the cause of death uncertainty in global health.

## Introduction

### How things are: The state of pathology in Global South

There is a rich body of literature on the duty and critical role of clinicians – frontline workers in saving lives in the Global South. There is however a gap in knowledge on the experiences of those who care for the dead in global health in times of crises and even during normal times. The last responders or ‘backline’ staff responsible for managing the dead bodies by retrieval, storage, burial, and or most importantly, determining and recording the cause of death, forms a critical part of their work. Such a significant role seems to be invisible in the global health discourse.

Data about pathology in the Global South is fragmented and incomplete, further contributing to the ‘invisibility’ of pathologists. Overtly, this information gap reflects and affirms the view that the discipline and practice of pathology in global health are often shunned upon and marginalised. According to Lancet’s special series on ‘*Pathology and laboratory medicine in low-income and middle-income countries’,* pathology does not attract funding from governments or donors and is overlooked compared to other disciplines (Horton et al., 2018; Sayed et al., 2018; Wilson et al., 2018). Moreover, there is a shortage of trained pathologists in global health. In sub-Saharan Africa, the number of anatomic pathologists is approximately one per 1 million patients (Wilson et al., 2018). In Ghana, there are only 20 qualified pathologists and their workload is skewed towards undertaking medico-legal work (Anim, 2015).

Further, pathologists are reported to be invisible in national and international health discourse (Horton et al., 2018; Oluwasola et al., 2009; Sayed et al., 2018; Wilson et al., 2018) and the level of awareness among policymakers of the importance of pathology is insufficient (Fleming et al., 2017; Horton et al., 2018; Oluwasola et al., 2009; Sayed et al., 2018; Wilson et al., 2018). This has caused a gap in critical knowledge that has further dire implications for welfare. This is so because pathology plays a critical role in tackling pandemics (Lucas, 2021); furthermore according to (Wilson et al., 2018) ‘*inaccurate diagnosis and detection of disease, informing prognosis and guiding treatment, contributing to disease screening, public health surveillance and disease registries, and supporting medical-legal systems*’.

### Autopsy – a dying profession in Global South

Autopsies – the examination of a body after death carried out by pathologists, including opening the deceased person's body and inspecting organs – are recognised as the most comprehensive and complete method to determine the cause of death (Becher et al., 2004; Burton & Underwood, 2007; Geller, 1983, 1986; Goldman et al., 1983; Kuijpers et al., 2014; Lyon, 2004). Among other benefits, autopsies contribute to greater accuracy in vital statistics by improving the quality of death certification and the identification and explanation of emerging diseases (Shojania & Burtons, 2008) and provide closure to families (Burton & Underwood, 2007). However, acceptability and the uptake of autopsy in the Global South are poor. It requires trained pathologists, who are scarce in low-resources contexts. There is also a general reluctance to use this method because, in many locations, it is considered to undermine religious, cultural, and social beliefs about the way a dead body should be treated (Cox et al., 2011; Maixenchs et al., 2016; Ngwenya et al., 2017). For example, touching and interfering with the body might be disapproved of (Gurley et al., 2011; Maixenchs et al., 2016); and consenting to an autopsy has been described as psychologically distressing for grieving families (Kosemehmetoglu et al., 2007; Lishimpi et al., 2001; Mfutso-Bengu & Taylor, 2002; Tan et al., 2010). As a consequence, medical workers are not willing to ask the deceased’s next-of-kin to consent to such procedures (Burton & Underwood, 2007).

### Cause of death uncertainty in Global South

Studies show that there are ‘falling rates’ of autopsies globally. It is, however, most likely that autopsy rates in the Global South have never been high, contributing to the uncertainty about the cause of death in local populations (Espinosa-Brito & de Mendoza-Amat, 2017; Oluwasola et al., 2009; Zheng et al., 1998). The literature describes that in many low- and middle-income settings, individuals often die without having been attended to by qualified medical personnel and are frequently buried or cremated without a documented medical history, and many times these deaths are not recorded at all (Abouzahr et al., 2015; Jha, 2014; Setel et al., 2007; Vogel, 2012). The resulting lack of reliable data and uncertainty around true causes of death leaves national and global health stakeholders without a good sense of how many people die in the world and from what causes (De Cock et al., 2019). This in turn hampers effective national health programmes and the most effective way of disbursing global health funding (Byass, 2009, 2016).

In lieu of the challenges highlighted in the preceding paragraph, and as a partial solution to address the knowledge gap, the World Health Organisation (WHO) has recommended the use of verbal autopsy – a structured interview carried out by a trained professional with relatives or other caregivers to estimate the cause of death when death occurs outside a health facility (WHO, 2016). A verbal autopsy has been utilised in many countries and has contributed to narrowing the information gap, however, it has many limitations. For example, although a verbal autopsy provides an estimation of the patterns of a cause of death at a community level, it is not an accurate method for attributing causes of death at the individual level (Byass et al., 2010). Furthermore, the verbal autopsy tool is not precise when determining the cause of death in children as the syndromes are often indistinguishable (Coldham et al., 2000; Soofi et al., 2015). In recent years, a new approach – minimally invasive autopsy (MIA) has been proposed as a solution to address the cause of death uncertainty. MIA involves using hollow needles to collect samples from vital bodily organs and so is argued to potentially be more acceptable and less invasive than a complete autopsy (Byass, 2016).

Drawing from the interviews conducted as part of a qualitative study to investigate the introduction of minimally invasive autopsy in the Global South, this paper explores the value of ‘body work’ (Twigg et al., 2011) performed by last responders charged with the duty of dead body management, with a focus on the Global South. In particular, this paper discusses the emotional, ethical, social, and political challenges pathologists face in ‘working on the bodies of others’ (Twigg et al., 2011) while conducting any form of post-mortem procedures – necessary for ascertaining and recording the causes of death.

## Methods

The findings of this paper are drawn from the interviews conducted as part of a qualitative study to investigate the perceptions of and ethical issues arising from the implementation of MIA technology in the Global South. While the study’s main focus was MIA, however the key findings emerging in the study were related to the challenges faced by the pathologists in the context of a dwindling pathology field in the Global South.

Data collection was conducted between January and November 2018. The key methodological tools employed in this project were semi-structured, in-depth interviews. This method enabled the collection of in depth-information by asking clarifying questions and requesting more in-depth accounts of the reasons behind respondents’ initial answers (Blaxter et al., 2006). A purposive sampling strategy**,** involving deliberately choosing respondents in order to reflect some features or characteristics of interest (Blaxter et al., 2006) was combined with a ‘snowball’ sampling. This sampling strategy was best suited to capture a wide range of voices, map out the range and different types of relevant experiences, and allowed sampling to be informed by the concurrent data analysis.

Forty-seven interviews were conducted with pathologists, a range of senior researchers, global health experts, healthcare professionals, and social scientists from the Global South and Global North. Many of the participants were key experts in their field, holding senior positions and possessing significant professional credentials, and they also described themselves as having several institutional affiliations. Participants’ sociodemographic data or the countries of participants work is not presented in order to maintain confidentiality.

Interviews were conducted face-to-face where possible, and otherwise via video-calling facilities or by phone, given the diverse geographical spread. The consent forms and information sheet explaining the aims and rationale of the project were sent in advance to all participants interviewed via Skype (or telephone) before the interview. The interviewees were asked for permission to be recorded. In the two instances where interviewees did not want to be recorded, notes were made during and immediately after the conversations. The remaining interviews were transcribed ‘word for word’. The study was approved by the Oxford Tropical Research Ethics Committee (OxTREC Reference: 521-17) as a minimal risk study.

Data analysis was an iterative process during the project, conducted in tandem with data collection. The interview data were analysed by using thematic analysis that allowed the mapping out of the issues, descriptions, interpretations, and experiences of the experts who were interviewed in the study. The analysis of the data started by reading transcripts of the interviews and getting familiar with the data set, making summaries of the themes appearing in each interview along with the recurring issues, novel findings, and divergent opinions of the research participants. Following the first few interviews, a preliminary exploratory analysis was conducted; that is, the data was initially hand-coded on printed documents by labelling fragments of interview extracts with phrases that captured the meaning of the data. The raw data (interview transcripts) was then entered into NVivo, but uncoded. The codes initially generated from the previous round of coding on interviews were entered as nodes. This produced an initial code list. Entering the data into NVivo allowed for further collapsing and merging of codes into initial themes. These were again recorded by going back to the interviews as part of an iterative process to check conceptual validity within codes. During the later recording steps, there were occasions when codes had to be revisited and re-categorised (Clarke & Braun, 2017). Finally, the codes produced after this round of NVivo recording were collapsed and resulted in the themes and sub-themes.

## Results

### Pathology policies in the Global South – ‘we do not fund this’

This research has identified several challenges faced by the pathologists, including challenges with a spillover effect, which contributes to the cause of death uncertainty in global health. The interviewees agreed that the pathology field which is central to diagnosing diseases for the living and cause-of-death determination has not attracted funding from governments or global health donors and has been overlooked in comparison to other medical disciplines and practices such as public health, internal medicine, or obstetrics. There have been suggestions that, at an institutional level, the pathology field and post-mortem procedures overall are being undervalued by funders and healthcare authorities, that is to say, that policymakers see little value in ‘budgeting’ for the dead. Some interviewees reported that there are attempts to strengthen the pathology infrastructure. However, those initiatives were perceived as ‘vertical’ interventions driven by the Global North donors addressing research questions or tackling a particular disease rather than building the human and institutional capacity in low-resource settings. Therefore, those efforts remain unsustainable and fragmented.

Pursuing pathology as a profession was also described as socially undesirable in some countries owing to the social stigma associated with the autopsy procedure and the way death and being around the dead is perceived. Moreover, the field of pathology has not been attracting many future specialists, this is in addition to the atrophy of institutional knowledge as older generations of pathologists retire. South Africa, for example, although equipped with the best pathology departments on the African continent, has suffered from the reality that its physicians are not sufficiently exposed to autopsies during their medical training, as an interviewee from South Africa claimed. This was seen to have created a disregard for the value of autopsies and a sense that they were superfluous; and has resulted in a lack of facilities for carrying out autopsies and the availability of a miniscule number of trained pathologists in a low resource context. For more reason, some interviewees strongly aver that pathologists need to be more proactive because:

We as a pathology discipline and as a medical discipline must promote post-mortems, we must find out where the barriers are and we must overcome them. I think that knowledge is so widely available to the lay public that in fact that information actually makes people buy-in rather than not. I don’t think there is anything to hide. We must be open and we must be inclusive and we must embrace it. (Global South, Pathologist)

### Physicians ‘breaking the chain’ of post-mortem requests – inaccurate data or no data

There are problems at the systemic and structural levels when it comes to postmortem because even doctors often do not see the need to request autopsies; talk less of distrustful families who were rarely consenting – ‘a vicious circle breaking at many points’, pathologists reported. In some places, the paperwork needed to carry out the procedure was reported to be onerous, that physicians had no incentive to request it unless they were particularly interested in the results. By not acknowledging the need for a post-mortem examination, physicians were ‘breaking the chain’ of post-mortem requests early in the process. This impasse, as some interviewees admitted, was also blamed on the pathologists. The results from the autopsy procedure in some locations were reported to be delayed, not robust, or even wrong due to a lack of resources or training; hence, they were not useful or trusted overall, as one interviewee claimed.

They [the pathologists] have got to convince their colleagues in obstetrics, paediatrics, medicine and surgery, and the Minister of Health, that pathology actually matters, and they have not done that. The system has worked not very well for decades without good pathology, so what’s the point? And it has got to be right, so there have to be proper controls, and they are not doing that. That is the problem. […] So, given that the service for the living people in terms of biopsies is not good, why would anybody be interested in the dead? It is even further removed; it is further off the horizon. That is the central point. (Global North, Pathologist)

Conversely, in many instances, either in a clinical setting or in research projects, pathologists were able to demonstrate discrepancies between clinical and post-mortem diagnoses highlighting the relevance of the autopsy procedure. The difference was explained to have resulted from deficiencies in the diagnostic site. Due to this puncture in the pathology process, doctors in many hospitals were left shorthanded or ill-equipped to answer some clinical questions and determine the cause of death. The ripple effect of such paucity of information is underscored to extend to the actual reporting of the cause of death. Some physicians made death certification in a manner that was described as ‘making an estimate about the most likely cause of death’ or even ‘guessing’ the cause of death. A case in point, some interviewees discussed some ethical issues with reporting the cause of death in the context of the HIV epidemic and how it impacts the accuracy of cause of death data.

What happens is it is usually a junior doctor who is asked to complete the death certificate, who looks through the clinical notes and makes a judgment as to what the most likely cause of death was and what the underlying causes were. So, they fill in what the cause of death was and what the underlying causes would be and that is based on all the diagnostic tests that are done ante-mortem. I think that’s going to be correct when patients have had a lot of time for clinical investigation prior to the death, but often patients present very sick and there is not enough time, so it is quite crude as to what is put on the death certificate. (Global South, Global Health Professional, Clinical Researcher, Infectious Diseases)

To further illustrate, some interviewees suggested that confusing the underlying cause of death with the immediate cause of death has resulted in an underreporting of HIV/AIDS mortality statistics in South Africa, which might result in misdirected clinical efforts. In the absence of autopsies, the pathologists interviewed stressed an uncertainty around the process for certifying the cause of death that feeds into the mortality statistics or missed opportunities to find out about emerging epidemics.

So, if you are sitting in a hospital, your issue is what do I have to treat here and now? That person is getting antiretrovirals and that is all fine, but I have to treat their pneumonia or whatever it is, and they may have TB as well, but their pneumonia is what has exacerbated [it]. So, when they come to write the cause of death, someone will page through and say ‘What was wrong? Ah pneumonia’. So, pneumonia is the cause of death. And so, you are getting misclassifications at every level. That’s a real pigsty of cause-of-death data. (Global South, Clinical Researcher, Infectious Diseases)

### The body being ‘mistreated’

The body work of pathologists conducting autopsy focuses directly on the bodies of the deceased. Although the body is dead, it is still closely identified with the person when alive. The body work, therefore, may have significant implications for the living, including the family members of a dead person who perceived themselves as representing the best interest of their loved one, the interviewees mentioned. For example, in a south-east Asian country, there were cases of families trying to put pressure on pathologists – who were carrying out their legal duties – to limit cuts and leave minimal incisions. In other cases, there were allegedly attempts to bribe the police to avoid an autopsy. Many interviewees working in Global South reported a high level of misperception and a lack of understanding of the autopsy procedure in their respective communities, contributing to the ambiguity or lack of trust towards the procedure, as illustrated in the quote below:

I think there is a lot of assumption when an autopsy was done that all the organs were replaced in the body and the body is returned to the family. I think there is a huge level of misperception or lack of understanding around exactly what happens and I am not sure that, especially for post-mortem purposes, is that with a grieving family, it is even more difficult to give adequate information and for them to fully process what you are telling them because of the emotional impact of the loss is so great. It is hard enough to get consent in a regular setting but to get consent in the context of bereavement it compromises the consent process. (Global South, Bioethicist)

Autopsy remains a taboo topic, as it is also associated with organ theft in some places or body mutilation. Interviewees reported that there is a high level of anxiety among families and communities about what they believe to be a ‘mistreatment’ of dead bodies that sometimes occurs inadvertently during some autopsies. Interviewees spoke about body parts, tissues, and organs having a symbolic significance shaped by religion, culture, and traditional knowledge systems. Some interviewees said that the body was perceived as sacred in their respective communities, had to be handled as little as possible, and should be buried or cremated intact. Therefore, trying to persuade families to consent to complete autopsies was often made difficult by the belief system related to the meaning of death and the practices related to death and burial.

There is also the cultural issue in Africa around traditional knowledge systems and the significance of body parts, you would get sometimes in a newspaper ‘Missing children body parts’ they would find a mutilated body and certain body parts are missing and those are being used for traditional practices, what they call traditional medicine. So as I say the belief system is quite entrenched. And especially when it happens in the setting of a criminal event where a child is abducted, goes missing, and the body is later found with parts missing, it also creates lots of distrust and anxiety. (Global South, Bioethicist)

It is against this background that some of my interviewees presented new approaches such as minimally invasive autopsy (MIA) to address anxieties about mutilation, as discussed in the quotation below:

It [MIA] is more socially acceptable because there is no disfigurement to the patient and when they [the family] see what you are doing – that you are pushing in a needle, a small core needle; you describe that you are going to make a tiny incision that is less than 1cm and take out a small core of tissues – that is very reassuring to them. This is much better because you are not opening the body up. Most adults have had some procedure or been exposed to a procedure where a needle is inserted into their body. So, there is a far greater acceptance and also it is rapid. (Global South, Clinical Researcher, Infectious Diseases)

### Social meaning attached to different bodies

There are social meanings pathologists, families and other stakeholders attach to different bodies. For example, a pathologist from a sub-Saharan country explained that the age of the deceased (too young or too old) determines if a family would give or withhold consent on the performance of an autopsy on their loved one. Furthermore, another pathologist from a Southeast Asian country discussed sensitivities around disclosing suicide as a cause of death; suicide in many locations is a taboo. To avoid the stigma associated with suicide, families put pressure on the certifying doctor in an attempt to conceal the death by suicide. The same pathologist discussed the status of dead bodies and suggests that it determines how they will be managed or treated. For example, dead bodies of illegal immigrants abandoned newborn babies were usually not accorded priority in police investigations owing to resource constraints and a perceived lack of importance, as illustrated in the quote below:

[A] very unpleasant situation **is newborn babies thrown somewhere**. They are not named, they are not citizens, they are not children of the country and in many cases, they come to our centre […] So in different conditions, some decompose. They are found on riversides. They have malaria. **The police are not very bothered about who gave birth to them, who are the mothers**. [They are] not going to trace them. [It is] not easy to trace all the time because the migrant population [in PLACE] is so huge. (Global South, Pathologist)

Some of the pathologists and health practitioners interviewed also reported on an ethical conundrum – finding a balance between the right of the deceased to withhold information about their health status beyond their physical death and the interests of others. For example, in the context of the social pressure around HIV/AIDS status, the underreporting of deaths from HIV/AIDS may be an active choice on the part of the certifying doctors. No pressure from families on the certifying doctors was mentioned by any of my interviewees; indeed, one participant, quoted below, reported that ‘there is implicit understanding’ between doctors and families about the process.

The doctor feels the family will be happier with a diagnosis of TB rather than HIV. So even there, just in the way it is reported, TB is the leading cause of death, [even though] it is not the leading cause of death. I think there is an implicit understanding. I don’t know if they negotiate [[…]] I suspect there is more of an implicit societal acceptance that we can’t – this person has just died – we can’t condemn them to have HIV for the rest of existence. (Global South, Clinical Researcher, Infectious Diseases)

### Importance of time

The temporal aspect of autopsy body work is central to its provision (Twigg et al., 2011). In some low-resource settings, mortuary facilities experience power cuts or limited storage spaces, and the post-mortem procedure needs to be conducted promptly to prevent the decomposition of the body. In addition, as one of the pathologists reported, families often pressure pathologists to release the body to proceed with timely funeral arrangements. For Muslims for instance, it is stipulated that the body should be buried within 24 h of death as a show of respect for the deceased, as some interviewees affirmed. In other cases, an autopsy procedure might interfere with transport arrangements for the body if such arrangements have already been made, especially when the body needs to be transported to a remote place such as the home village of the deceased. The delay, as illustrated by the quote below, may be caused by the deliberation process among the family members whether or not to consent to the procedure.

In the African tradition often the decision is a collaborative decision, so your husband or wife dies and you say I am allowing this person. You gather the family and you have a discussion because it is a different way of existing, you don’t exist in isolation you exist within a larger family group and they are all part of your life. So often there is more, so there are time delays, you wait for family members to arrive. And then also it is when funerals can be performed if someone dies and the family feels that the most appropriate time is this coming weekend, well then, that’s it. (Global South, Pathologist)

Some interviewees also suggested that in some cases, requesting a hospital autopsy procedure was vehemently opposed by the locals running the hospitals and private mortuaries. Their argument for such refusal is based on the practicality that the process will delay a funeral and result in financial loss. Thus, the time factor pertaining to the post-mortem creates ‘a spoke in the wheels’ when it comes to handling the dead. As one interviewee puts it, there was ‘a perverse incentive’ to avoid carrying out an autopsy.

## Discussion

### Pathologists: Invisible back-room people

**Figure 1.**
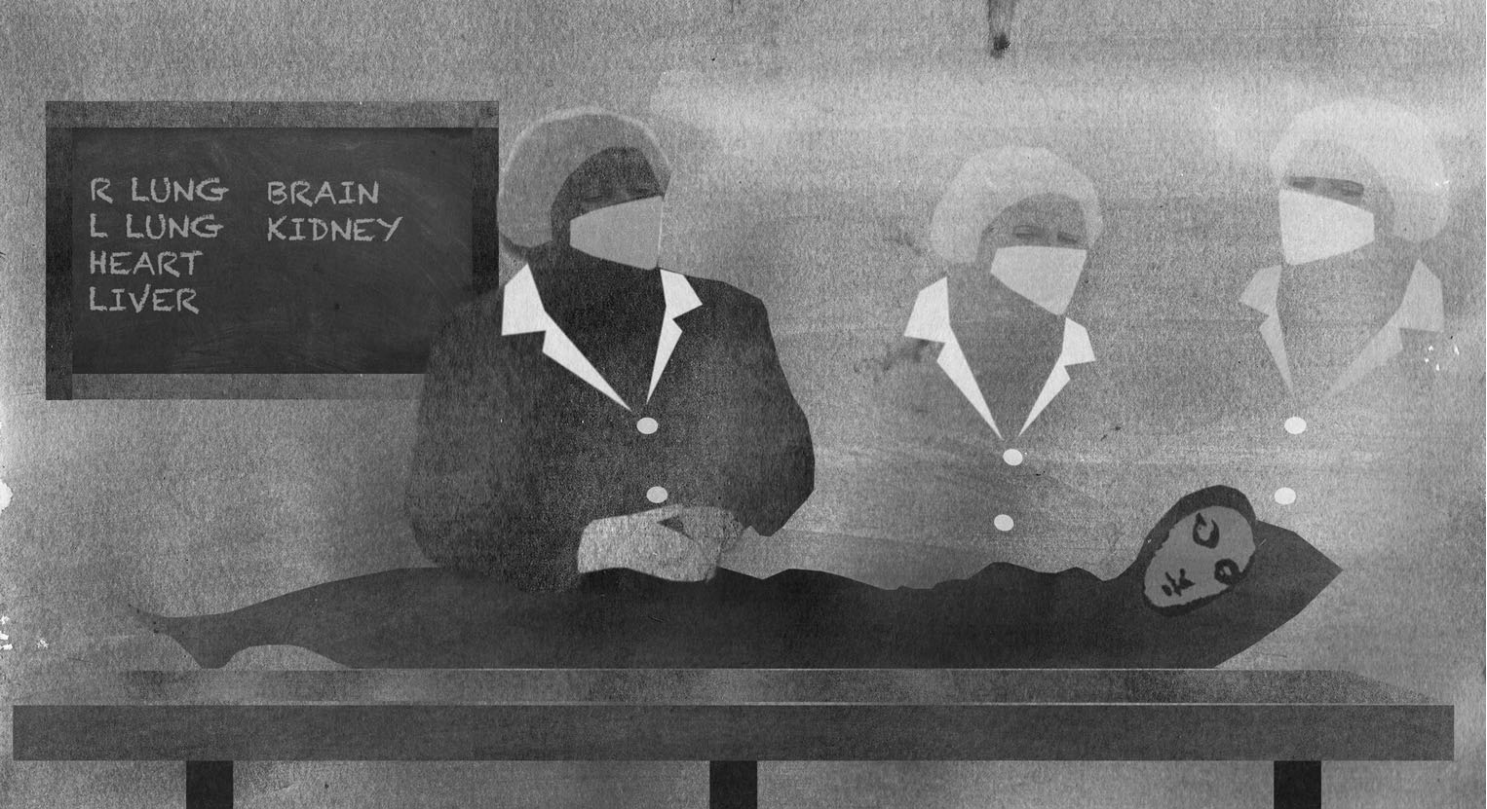
Pathologists have described themselves as ‘backroom people’ or ‘invisible’ Credit: Credit Anna Suwalowska. Copyright © Anna Suwalowska (2022).

Pathologists, despite their critical role, see themselves as ‘backroom people’ (see Figure 1) because their work happens behind the scenes with no actual contact or engagement with the family of the deceased. Even the locations of mortuaries in some instances also create a physical distance from colleagues and families alike. More than that, post-mortem duties are conducted in the ‘bowels’ of hospitals, in remotely located mortuaries, or in designated areas; and that contributes to how their critical role is further shrouded in invisibility as a consequence. The dead body is central to their activities; but because of the invasive nature of the autopsy procedure, certain cultural beliefs about the impurity of ‘dead body work’, symbolic or literal distance is forced between pathologists, other healthcare professionals, and the general public. Essentially, the ‘invisibility’ of pathologists and underlying lack of knowledge or misconception, about post-mortem procedures has contributed to social uncertainty and created potential ethical dilemmas for families worried about ‘the right thing to do’ – concerns that are also present in the acceptance of all post-mortem procedures. This invisibility also creates other ethical and social tensions such as the lack of trust and suspicion about postmortem among families, which in turn results in low autopsy rates. Against this background, some studies called for more engagement with the family members to better understand their concerns and also for more action to educate about the value of postmortem (Banyini et al., 2013; Lishimpi et al., 2001).

### The micro-politics of the body work interaction

This study found that micro-politics of the body work interaction, particularly how the institutional power of healthcare professionals is embodied through interactions (Twigg et al., 2011) points to a reality where pathologists consider themselves to be the only ones who understand the value of post-mortems. In a similar vein, some clinicians were described as being notably sceptical about conducting autopsies in settings where resources are limited. They are rather interested in deploying these scarce resources to diagnose and treat living patients. This finding resonates with earlier studies that have made a case for improving this knowledge, especially in medical settings. It has been suggested that clinicians should receive training on the value of autopsy for their clinical practice so that they request it more frequently (Echo et al., 2015; Lishimpi et al., 2001 Pawar & Pawar, 2012;). The literature agrees that clinicians may be reluctant to request autopsy procedures because they anticipate a difficult conversation with bereaved family members and a refusal to consent. Lishimpi et al. (2001), for instance, explained that medical staff is ‘unsupported’ and ‘unprepared’ to request consent for an autopsy, which is the ‘most difficult task’. If that is the case, further research is needed to investigate the challenges faced by clinicians in requesting autopsies and whether more interactions between pathologists and clinicians would improve the perception of pathology.

### Social person in the corpse

Although the body is dead, the social person is still present in the corpse (Twigg et al., 2011) representing ‘an array of built-in memories that can never be completely separated from it (Jones & Whitaker, 2009). The dead body, therefore, has ethical significance to the living. What is done to the dead body is of concern to the family members who want the body of the loved one to be treated with dignity and with respect to their religion and cultural practices. However, carrying out autopsies is widely viewed as taboo, raising some social and ethical tensions. Relatives fear the mutilation or disfigurement of the dead body and therefore reject the autopsy procedure (Oluwasola et al., 2009, Charlier et al., 2013). Some of the other reasons provided for unwillingness to give consent for autopsy were explained by a perception that the body is being commodified (Banyini et al., 2013), the body is being desecrated (Charlier et al., 2012), or there have been expressions of religious and cultural factors as the basis of objections to post-mortem procedures (Banyini et al., 2013; Echey et al., 2015; Yawson et al., 2014). Against this background, an autopsy is a sensitive issue for many people, and consenting to it has been described as psychologically distressing for grieving families (Mfutso-Bengu & Taylor, 2002). Notwithstanding the scientific benefits associated with the autopsy procedure, the concerns expressed by the family members should be prioritised, and their cultural and religious beliefs respected. Furthermore, more research is needed into the acceptability of the clinical autopsy procedure in the Global South and the ethical considerations associated with it, to better understand the complexities.

### ‘Scandal of invisibility’ – the body work of pathologists in a wider social and political context

Unlike other body work that involves ‘direct, hands-on activities, handling, assessing and manipulating bodies’, the body work of pathologists is situated within a wider social, political, and economic trends (Twigg et al., 2011). Autopsies are rarely conducted in low and middle-income settings, and its utility is limited to research projects funded by the Global North or forensic investigations. The dearth of autopsy procedures in the Global South has been identified by many as an important factor that contributes to uncertainty about the causes of death. Mortality patterns for the majority of the world's population remain unrecorded, representing a major constraint on global health and development. The information gap also has significant ethical consequences. Lancet named this situation a ‘Scandal of invisibility’ – where most of the world's poor are unseen, uncountable, and hence uncounted (Setel et al., 2007). Global and national stakeholders have been devising alternative solutions to collect mortality data through, a case in point is a relatively new initiative established to address child mortality – Child Health and Mortality Prevention Surveillance Network (CHAMPS) Program funded by the Bill and Melinda Gates Foundation (Foundation, 2015). In many places where mortality is high, there is no capacity to understand ‘what is killing people’. Therefore CHAMPS and other similar programmes could provide capacity development to strengthen local pathologists and pathology systems, including having a good histology laboratory service for conducting laboratory tests and instant pathology examinations that countries would need as they move forward with building their healthcare systems. Finally, as discussed in the Lancet series, pathologists have been not advocating for their own work in their countries and also globally. This paper recommends they should start doing so by sharing their knowledge and advocating for their discipline.

## Conclusion

This article has explored the value of ‘body work’ performed by the last responders charged with ascertaining and recording the causes of death. In particular, this paper has discussed the emotional, ethical, social, and political challenges pathologists face in ‘working on the bodies of others’ while conducting any form of post-mortem procedures – necessary for determining the cause of death. This paper has demonstrated that adequate management of a dead body signifies respectful treatment of the dead and more, the resultant mortality data has significant consequences for the living. The field of pathology, however does not attract funding from governments or donors and is overlooked compared to other fields. This study has also shown that the work of the pathologists is often unrecognised and ‘invisible’ not only to the policymakers but also to their fellow clinicians and the families who either do not see the value of conducting an autopsy procedure or have very limited knowledge about the procedure.
